# Supplementary material for: Ultra-fast and accurate electron ionization mass spectrum matching for compound identification with million-scale in-silico library
Source: Nat Commun. 2023 Jun 22;14:3722. doi: 10.1038/s41467-023-39279-7 (PMC10287733; doi:10.1038/s41467-023-39279-7)
Supplement: Supplementary file 1 — Supplementary Information [file 41467_2023_39279_MOESM1_ESM.pdf]

Supplementary Information

Ultra-fast and accurate electron ionization mass spectrum matching for compound identification with million-scale *in-silico* library

Qiong Yang[1,†], Hongchao Ji[2,†], Zhenbo Xu[1], Yiming Li[1], Pingshan Wang[1], Jinyu Sun[1], Xiaqiong Fan[1], Hailiang Zhang[1], Hongmei Lu[1, *], Zhimin Zhang[1, **]

1. College of Chemistry and Chemical Engineering, Central South University, Changsha, 410083, PR, China
2. Agricultural Genomics Institute at Shenzhen, Chinese Academy of Agricultural Sciences, Shenzhen, 518120, PR, China

†. These authors contribute equally.

[*] Corresponding Author: hongmeilu@csu.edu.cn

[**] Corresponding Author: zmzhang@csu.edu.cn

Table of Contents

Supplementary Figures 2

Supplementary Fig. 1. Details of the datasets, libraries, and Word2vec model construction. 2

Supplementary Fig. 2. Distribution of WCS between the predicted and experimental spectra in the test set. 3

Supplementary Fig. 3. Venn diagram of the compound classes in the *in-silico* library and the expanded library. 3

Supplementary Fig. 4. Venn diagram of the recall@1 molecules of FastEI and WCS. 4

Supplementary Fig. 5. The cosine similarity difference between the spectral binning vectors and Word2vec embeddings. 4

Supplementary Fig. 6. Five-step filtering procedure. 5

Supplementary Fig. 7. UMAP plots of different representations of the experimental spectra in the test set. 5

Supplementary Fig. 8. The spectrum matching performance of **a** recall@1, **b** recall@10, and **c** time on the validation set for various M, and *ef*Search (*ef*Construction = 600) parameters. 6

Supplementary Table 7

Supplementary Table 1. Optimization of hyperparameters of the Word2vec model on the validation set. 7

Supplementary Notes 8

Supplementary Note 1. The weighted cosine similarity. 8

Supplementary Note 2. The experimental information of the measured spectra of the extra test set 8

Supplementary Figures

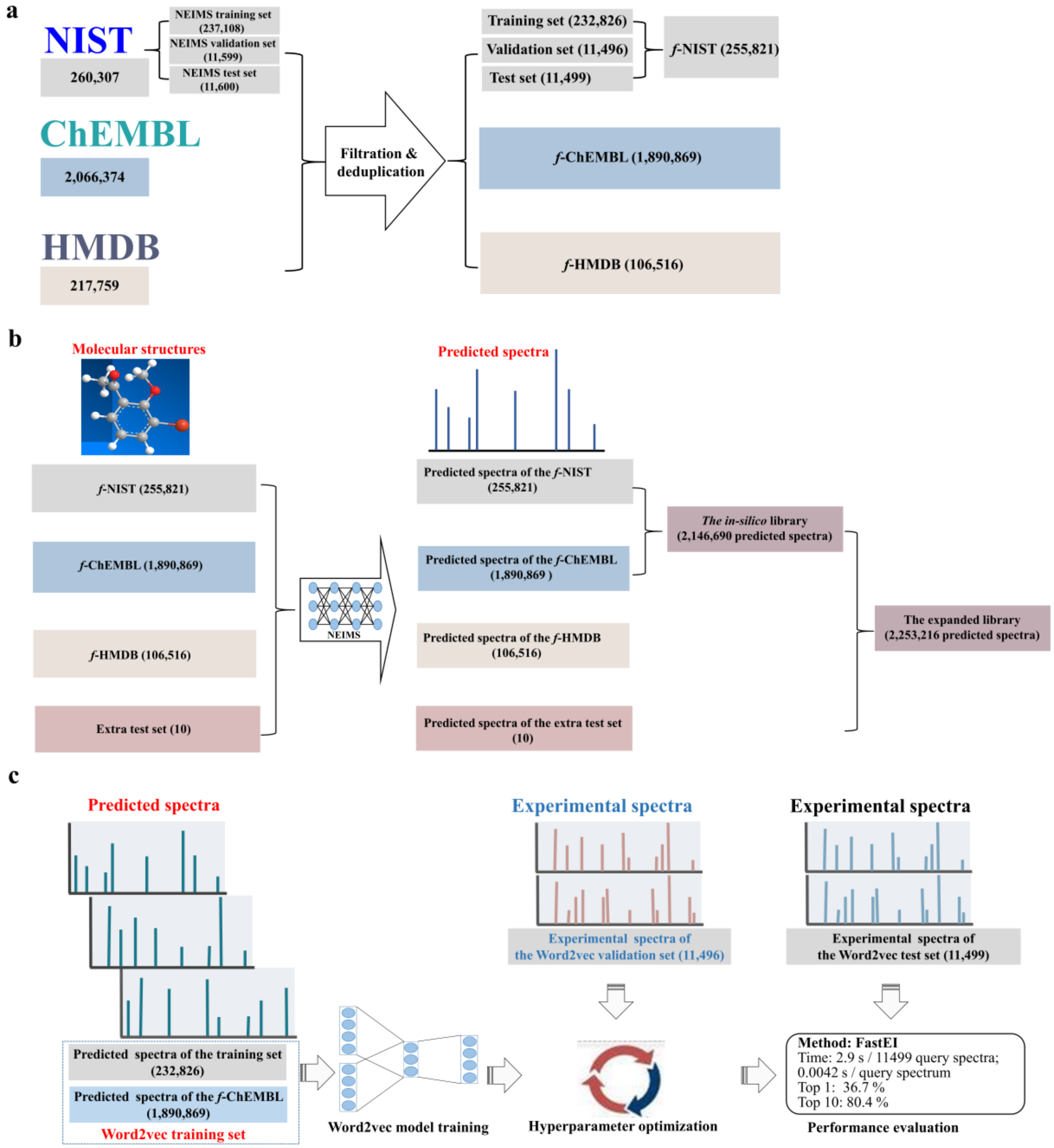

Supplementary Fig. 1. Details of the datasets, libraries, and Word2vec model construction. a Detailed information on datasets used in FastEI. **b** Construction of the *in-silico* libraries. **c** Building of the Word2vec model.

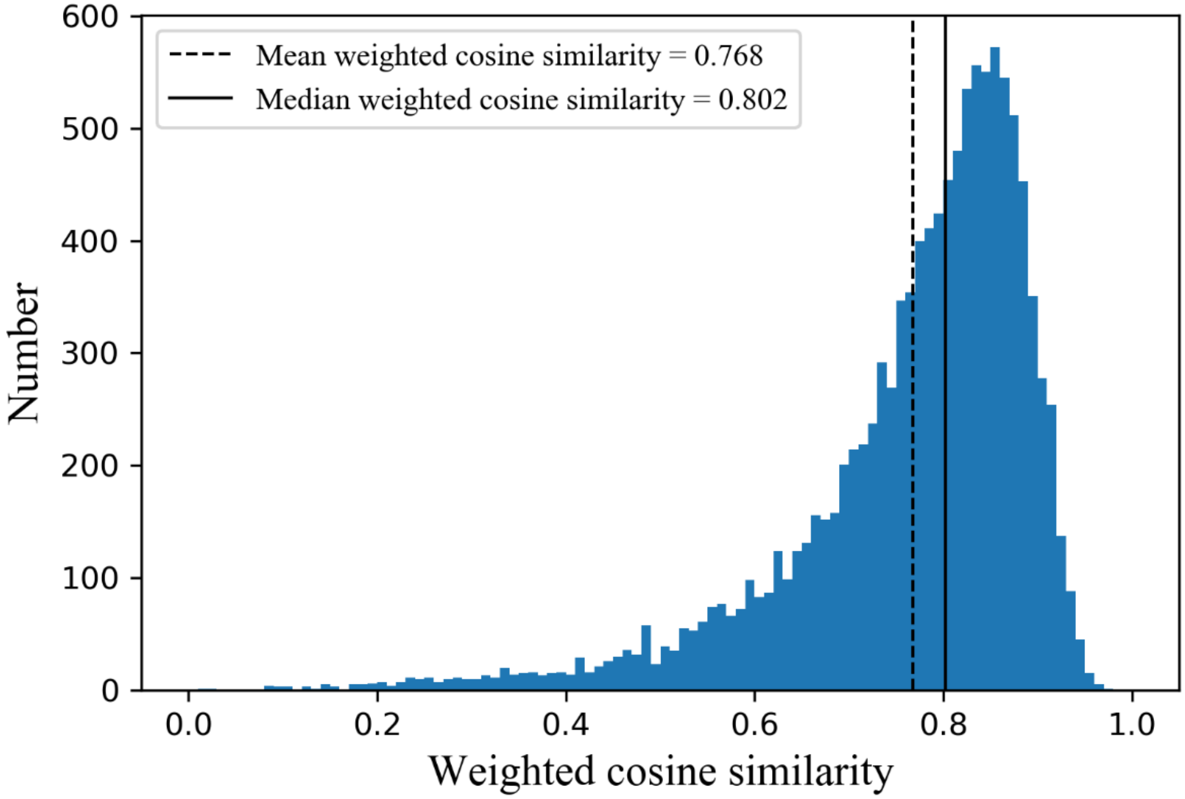

Supplementary Fig. 2. Distribution of WCS between the predicted and experimental spectra in the test set**.**

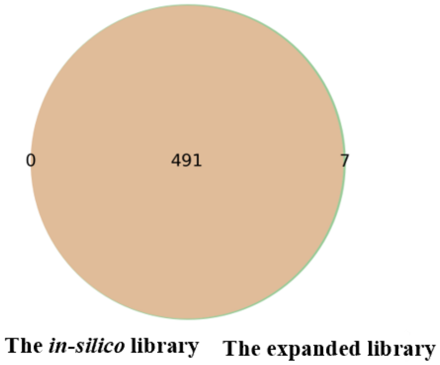

Supplementary Fig. 3. Venn diagram of the compound classes in the *in-silico* library and the expanded library. Seven new classes in the expanded library: Homogeneous metalloid compounds, Dioxoles, Oxirenes, Molybdopterin dinucleotides, Sulfines, Glycinamide ribonucleotides, Organosulfenic acids and derivatives.

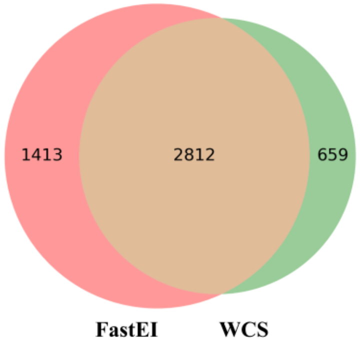

Supplementary Fig. 4. Venn diagram of the recall@1 molecules of FastEI and WCS. The experimental spectra (collected from the NIST main library) of 11,499 molecules in the test set were used to match the *in-silico* library by FastEI and WCS. The target molecules of the 2,812 spectra ranked top1 in the FastEI and WCS matching methods. The target molecules of the 1,413 spectra ranked top1 by FastEI, and ranked outside of the top1 by WCS.

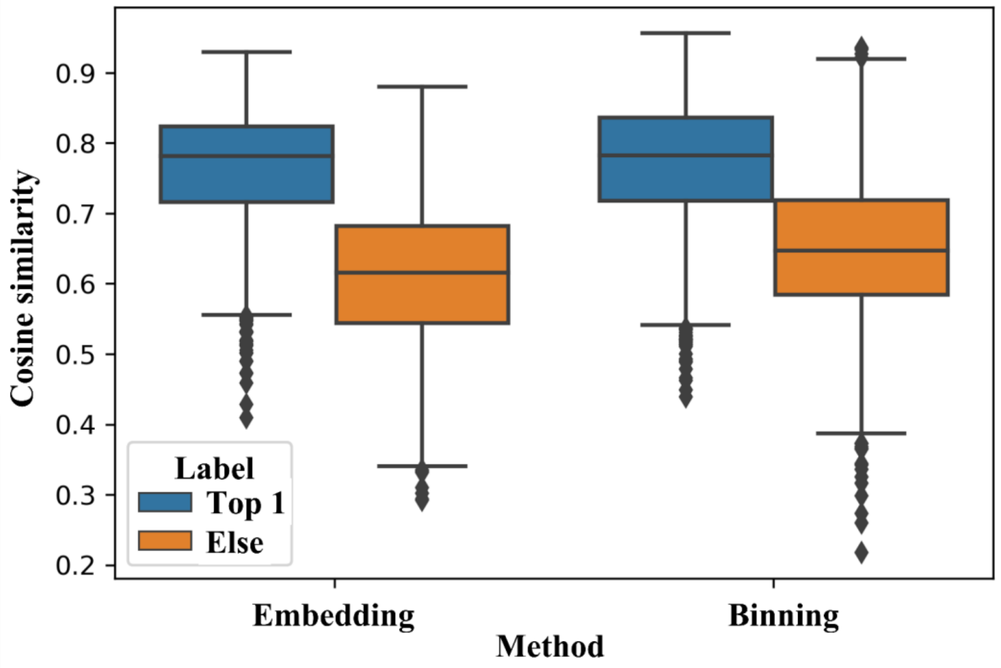

Supplementary Fig. 5. The cosine similarity difference between the spectral binning vectors and Word2vec embeddings. The predicted spectra of the top 10 candidates of these 1,413 experimental spectra matching results using FastEI are retrieved from the *in-silico* library. All the predicted and experimental spectra can be transformed into spectral embeddings by the Word2vec model. For each experimental spectra, the spectral binning similarity between experimental spectrum binning and its candidate predicted spectrum binning was calculated by the cosine similarity method. The embedding similarity between experimental spectrum embedding and its candidate predicted spectrum embedding was calculated by the cosine similarity method too. **Left**: box plot of the spectral embedding similarities of top1 candidates are compared with the embedding similarities of other candidates (the top10 except for top1); **Right**: box plot of the spectral binning similarities of top1 candidates are compared with the spectral binning similarities of other candidates (the top10 except for top1). It shows that the embedding similarity has better distinguishability than spectral binning similarity. It can be said that the embedding similarity can make the same molecules have high cosine similarity and different molecules have low cosine similarity.

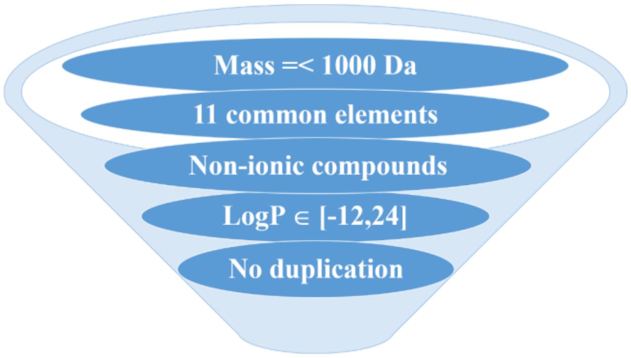

Supplementary Fig. 6. Five-step filtering procedure, including (1) molecular mass is less than 1000 Da; (2) molecules only contain 11 common elements H, C, O, N, P, S, Cl, F, Br, I, Si; (3) molecules are not ionic compounds; (4) molecular LogP is within the range from -12 to 24; (5) repeating molecules was deduplicated within the dataset.

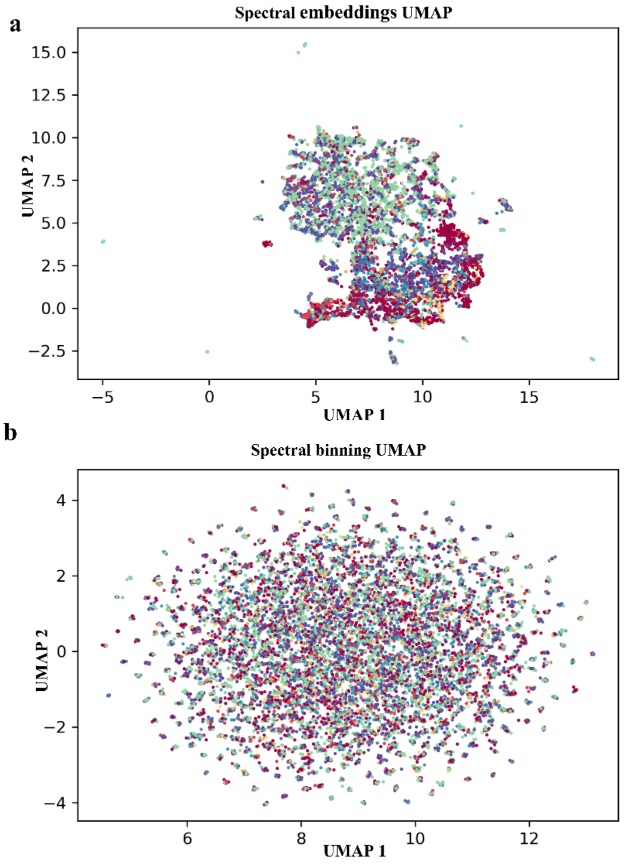

Supplementary Fig. 7. UMAP plots of different representations of the experimental spectra in the test set. ClassyFire has been used to classify molecules in the test set into 21 chemical superclasses. **a** the UMAP plot of Word2vec embeddings colored by the chemical superclass. There is a tendency for molecules to aggregate by their chemical superclasses in the UMAP space after Word2vec embedding. **b** the UMAP plot of spectral binning vectors colored by the chemical superclass. It can be seen that the molecules are randomly distributed in UMAP space and are unrelated to their chemical superclasses. It can be concluded that the Word2vec embeddings are more relevant to the chemical superclasses than the spectral binning vectors by comparing the UMAP plot **a** and **b**.

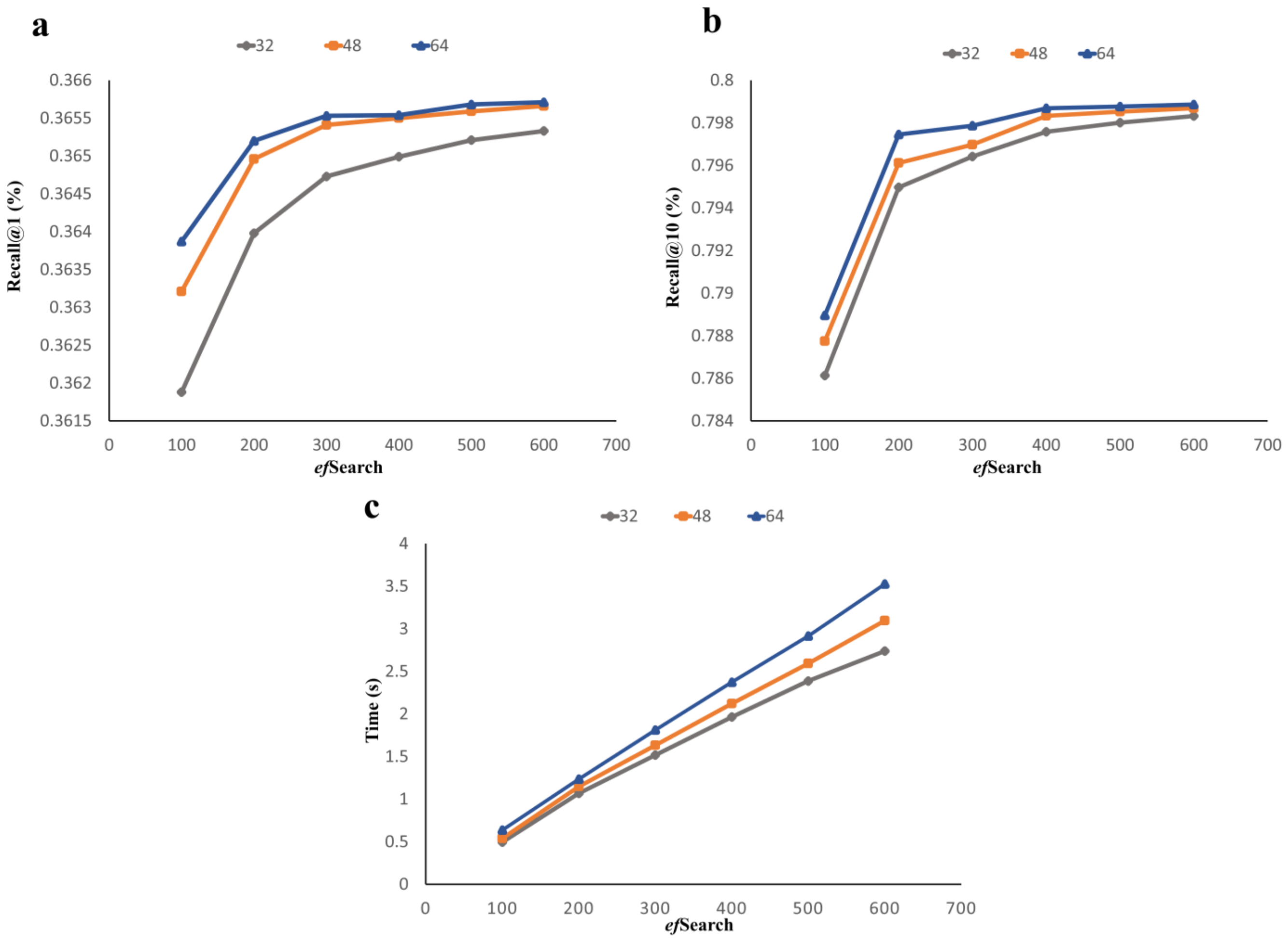

Supplementary Fig. 8. The spectrum matching performance of **a** recall@1, **b** recall@10, and **c** time on the validation set for various M, and *ef*Search (*ef*Construction = 600) parameters.

Supplementary Table

Supplementary Table 1. Optimization of hyperparameters of the Word2vec model on the validation set.

Key parameters	Value	Matching performance of the validation set		Explanation
		Recall@1 (%)	Recall@10 (%)	
Word vector dimension (epoch = 60)	200	30.5	72.6	
	300	35.4	79.0	--
	400	37.0	80.6	--
	**500**	**37.3**	**81.0**	--
	600	37.2	81.0	--
Epochs (dim = 500)	40	37.2	80.9	--
	**60**	**37.3**	**81.0**	--
	80	37.1	81.0	--
Window size	1000	--		The combination of all peaks is meaningful in mass spectra.
Negative sampling	5	--		Default
CBOW mode	sg=0	--		Refer to Spec2Vec
Initial learning rate	0.025	--		Default
Learning rate decay per iteration (per epoch)	0.00025	--		Default

Supplementary Notes

Supplementary Note 1. The weighted cosine similarity

The exact form of the weighted cosine similarity (WCS) is given below:

$$Similarity(I_q, I_l) = \frac{\sum_{k=1}^{M_{\max}} m_k I_{qk}^{0.5} . m_k I_{lk}^{0.5}}{\left\| \sum_{k=1}^{M_q} (m_k I_{qk}^{0.5})^2 \right\| \left\| \sum_{k=1}^{M_l} (m_k I_{lk}^{0.5})^2 \right\|} \qquad \text{S-1}$$

Here, I_q and I_l are vectors of m/z intensities representing the query spectrum and the library spectrum, respectively; m_k and I_k are the mass-to-charge ratio and intensity found at m/z = k; M_l and M_q are the largest indices of I_q and I_l with nonzero values; and $M_{\max}$ is the larger of M_l and M_q.

Supplementary Note 2. The experimental information of the measured spectra of the extra test set

The ten compounds in the extra test set were measured on a Shimadzu GC-2010 gas chromatography coupled with a Shimadzu QP2010Ultra mass spectrometer (Shimadzu, Japan), equipped with an autosampler GL 221-34618. The column used to analyze the organic samples was an Agilent DB-5MS with a deactivated fused silica column (30 m × 0.25 mm × 0.25 μm). Helium (99.99% purity) was used as carrier gas with a flow rate of 1.0 mL/min. One microliter of sample solution was injected in split mode with a split ratio of 1:10, and the temperature of the injector was 280 °C. The initial temperature of the column was 60 °C, and the optimized temperature program was held at 60°C for 1 min and then raised to 290 °C at 10 °C/min and held for 5 min. The total running time was 29 min, and the solvent was delayed for 2.8 min. The experimental conditions of the mass spectrometer were as follows: ion source and interface temperatures were 230 °C and 250 °C, respectively; the detector voltage was 0.8 kV, and data were acquired in full scan mode (29-500 m/z).
